# Identification of Small Molecule Inhibitors of the Deubiquitinating Activity of the SARS-CoV-2 Papain-Like Protease: *in silico* Molecular Docking Studies and *in vitro* Enzymatic Activity Assay

**DOI:** 10.3389/fchem.2020.623971

**Published:** 2020-12-08

**Authors:** Eleni Pitsillou, Julia Liang, Katherine Ververis, Kah Wai Lim, Andrew Hung, Tom C. Karagiannis

**Affiliations:** ^1^Epigenomic Medicine, Department of Diabetes, Central Clinical School, Monash University, Melbourne, VIC, Australia; ^2^School of Science, College of Science, Engineering and Health, RMIT University, Melbourne, VIC, Australia; ^3^Department of Microbiology and Immunology, The University of Melbourne, Parkville, VIC, Australia; ^4^Department of Clinical Pathology, The University of Melbourne, Parkville, VIC, Australia

**Keywords:** coronavirus, COVID-19, SARS-CoV-2, papain-like protease, deubiquitinase inhibitors, molecular docking

## Abstract

COVID-19 is an ongoing pandemic caused by the SARS-CoV-2 virus with important political, socio-economic, and public health consequences. Inhibiting replication represents an important antiviral approach, and in this context two viral proteases, the SARS-CoV-2 main and papain-like proteases (PL^pro^), which cleave pp1a and pp1ab polypeptides, are critical. Along with protease activity, the PL^pro^ possesses deubiquitinating activity, which is important in immune regulation. Naphthalene-based inhibitors, such as the well-investigated GRL-0617 compound, have been shown to possess dual effects, inhibiting both protease and deubiquitinating activity of the PL^pro^. Rather than binding to the canonical catalytic triad, these type of non-covalent inhibitors target an adjacent pocket, the naphthalene-inhibitor binding site. Using a high-throughput screen, we have previously identified the dietary hypericin, rutin, and cyanidin-3-O-glucoside compounds as potential protease inhibitors targeting the naphthalene-inhibitor binding site. Here, our aim was to investigate the binding characteristics of these compounds to the PL^pro^, and to evaluate deubiquitinating activity, by analyzing seven different PL^pro^ crystal structures. Molecular docking highlighted the relatively high affinity of GRL-0617 and dietary compounds. In contrast binding of the small molecules was abolished in the presence of ubiquitin in the palm subdomain of the PL^pro^. Further, docking the small molecules in the naphthalene-inhibitor binding site, followed by protein-protein docking revealed displacement of ubiquitin in a conformation inconsistent with functional activity. Finally, the deubiquitinating activity was validated *in vitro* using an enzymatic activity assay. The findings indicated that the dietary compounds inhibited deubiquitinase activity in the micromolar range with an order of activity of GRL-0167, hypericin >> rutin, cyanidin-3-O-glucoside > epigallocatechin gallate, epicatechin gallate, and cefotaxime. Our findings are in accordance with mechanisms and potential antiviral effects of the naphthalene-based, GRL-0617 inhibitor, which is currently progressing in preclinical trials. Further, our findings indicate that in particular hypericin, rutin, and cyanidin-3-O-glucoside, represent suitable candidates for subsequent evaluation as PL^pro^ inhibitors.

## Introduction

Coronavirus disease (COVID-19) was declared a pandemic on the 11th of March 2020 (World Health Organization, 2020). The first reported cases of this disease came from Wuhan, China in late 2019, and the infectious agent responsible for causing this disease was identified as severe acute respiratory syndrome coronavirus 2 (SARS-CoV-2) (Coronaviridae Study Group of the International Committee on Taxonomy of Viruses, 2020). Since the start of the year, the scientific literature on COVID-19 has increased and the findings from these studies have formed an integral part of the public health response.

In regards to treatment options, a number of vaccine trials have been established and there is also a focus on drug repositioning (Bar-Zeev and Moss, 2020; Folegatti et al., 2020). The U.S Food and Drug Administration (FDA) has recently approved remdesivir, which is an RNA-dependent RNA polymerase inhibitor, as a COVID-19 treatment for hospitalized patients (FDA, 2020). Interestingly, the WHO Solidarity trial has produced contradicting findings regarding the effectiveness of remdesivir (Pan H. et al., 2020). In addition to antiviral drugs, the efficacy of compounds that have immunomodulating properties are also being investigated (de la Rica et al., 2020).

Further research is required to establish the precise mechanisms of action of potential therapeutic drugs and determine their biological targets (Zhou Y. et al., 2020). The virus replication cycle is comprised of several stages and compounds that inhibit key proteins involved in these steps may have antiviral properties (Jeong et al., 2020; Pandey et al., 2020). Papain-like protease (PL^pro^) is a cysteine protease enzyme that is encoded by the multi-domain non-structural protein 3 (nsp3) and is required for polypeptide processing (Báez-Santos et al., 2015; Folegatti et al., 2020). The role of the SARS-CoV-2 PL^pro^ in viral replication and the regulation of the innate immune response is being explored (Shin et al., 2020).

Ubiquitin and ubiquitin-like proteins, such as interferon-stimulated gene 15 (ISG15), are important effector molecules of the antiviral immune response (Jiang and Chen, 2011; Perng and Lenschow, 2018). Through binding to target proteins, various cellular pathways can be modulated (Jiang and Chen, 2011; Perng and Lenschow, 2018). Viruses have developed mechanisms to evade detection and destruction by the host's immune response, and these strategies continue to evolve (Nelemans and Kikkert, 2019). In terms of SARS-CoV-2, the deubiquitinating and deISGylating activities of the PL^pro^ enzyme have been described (Bosken et al., 2020; Klemm et al., 2020; Rut et al., 2020; Shin et al., 2020). GRL-0617 is a naphthalene-based inhibitor that has been found to interfere with the protease, deubiquitinase and deISGylating activities of the SARS-CoV and SARS-CoV-2 PL^pro^ enzymes (Ratia et al., 2008; Freitas et al., 2020; Gao et al., in press). The SARS-CoV-2 PL^pro^ has consequently been identified as an attractive drug target and in this study, the deubiquitinase activity of this viral protein was of interest (McClain and Vabret, 2020).

The health-promoting properties of dietary compounds have been extensively explored over the years and in response to the COVID-19 pandemic, bioactive compounds are being investigated further (Dhama et al., 2020; Mani et al., 2020). There is a growing body of literature on the antiviral and immunomodulating properties of plant-based compounds, and their potential use as therapeutic agents against SARS-CoV-2 (Tiwari et al., 2018; Panyod et al., 2020; Tahir Ul Qamar et al., 2020). This includes traditional Chinese and traditional Indian medicinal compounds, vitamins, curcumin, glycyrrhizic acid, tea polyphenols and compounds derived from *Allium sativum* to name a few (Chen et al., 2020; Divya et al., 2020; Donma and Donma, 2020; Tripathi et al., 2020). Enhancing the bioavailability of natural compounds continues to be a challenge however, their structures can be used as scaffolds for the development of novel drugs (Ngwa et al., 2020).

*In silico* methods were used to compare the binding mode of naphthalene-based inhibitors (GRL-0617 and 3k) to dietary compounds including hypericin, rutin, cyanidin-3-O-glucoside and (-)-epigallocatechin gallate. The antimicrobial, anti-inflammatory and antioxidant activities of these phytochemicals have been reported and their structures may even be used as scaffolds in the drug development process (Mohammadi Pour et al., 2019). The aim was to determine whether the dietary compounds were able to bind in a similar manner as the positive control GRL-0617, and potentially interfere with the binding of ubiquitin. The results were validated further using *in vitro* assays.

## Materials and Methods

### Protein Structures and Ligands

Several crystal structures of the SARS-CoV-2 PL^pro^ were obtained from the RCSB Protein Data Bank (PDB ID: 6xaa, 6w9c, 6wuu, 6wx4, and 7jrn) (Berman et al., 2000; Klemm et al., 2020; Osipiuk et al., 2020; Rut et al., 2020; Sacco et al., 2020). The SARS-CoV PL^pro^ (PDB ID: 4mm3) and MERS-CoV PL^pro^ (PDB ID: 4rf0) were also used for comparison in this study (Bailey-Elkin et al., 2014; Ratia et al., 2014). Crystallographic waters were removed and the native zinc ions were retained. A ubiquitin chain was present in the structures of 6xaa, 4mm3, and 4rf0, which was used to generate two sets of docking data for each protein: PL^pro^ in complex with ubiquitin and, and apo PL^pro^ in the absence of ubiquitin. The ubiquitin in each complex was also isolated for protein-protein docking. The ligands that were used in this *in silico* study were the naphthalene inhibitors GRL-0617 and 3k, and the dietary compounds (-)-epigallocatechin gallate, cyanidin-3-O-glucoside, rutin and hypericin. The structures of the dietary compounds were obtained from the National Center for Biotechnology Information PubChem (Kim et al., 2019). GRL-0617 and 3k were drawn using Chem3D 19.0 (Perkin Elmer, Massachusetts, USA).

### Molecular Docking Using the Schrödinger Suite

Molecular docking was performed using the Schrödinger Suite (Schrödinger, 2020a). The protein structures were prepared using the Protein Preparation Wizard, while the compounds were prepared using the LigPrep tool (Madhavi Sastry et al., 2013; Schrödinger, 2020b). The default settings were used for both of these steps and the optimized potentials for liquid simulations 3e (OPLS3e) force field was selected (Jorgensen and Tirado-Rives, 1988; Jorgensen et al., 1996; Shivakumar et al., 2010; Harder et al., 2016). The top ranking ligand conformation was selected for the subsequent molecular docking stage.

The residues that were within 5Å of the co-crystallized ligand GRL-0617 in the 7jrn structure were used to generate the receptor grid for each SARS-CoV-2 PL^pro^ (Friesner et al., 2004, 2006; Halgren et al., 2004; Schrödinger, 2020c). These residues were E167, K157, Y273, D164, G163, L162, C270, Q269, Y268, N267, G266, Y264, P248, P247, M208, and T301. To determine the corresponding residues in the SARS-CoV and MERS-CoV crystal structures, pairwise alignment was performed in the Multiple Sequence Viewer tool (Schrödinger, 2020a). The residues used to form the grid for SARS-CoV PL^pro^ were E168, K158, Y274, D165, G164, L163, C271, Q270, Y269, N268, G267, Y265, P249, P248, and M209. For MERS-CoV PL^pro^, the receptor grid was generated based on the residues R1649, C1639, Y1760, D1646, D1645, P1644, V1757, A1756, T1755, E1754, G1752, F1750, P1731, T1730, V1691, and T1789.

The receptor grids were 20 × 20 × 20 Å in size and the OPLS3e force field was utilized. The compounds were then docked to each protein structure using the quantum-polarized ligand docking (QPLD) protocol, as previously described (Liang et al., 2020).

### Blind Docking and the Prediction of Ligand-Binding Sites

The PL^pro^ crystal structures and the compounds were prepared as macromolecules and ligands in PyRx, respectively (Dallakyan and Olson, 2015). The corresponding.pdbqt files were obtained and the receptor grid was generated around the entire surface of the protein. The exhaustiveness was set to 2048. AutoDock Vina was used for molecular docking and the jobs were run on the cloud-computing server Galileo (Hypernet Labs) (Trott and Olson, 2010; Hypernet Labs Galileo, 2020). In addition to blind docking, the PrankWeb server was used to identify potential binding pockets that were conserved in the 6xaa, 4mm3, and 4rf0 crystal structures (Jendele et al., 2019).

### Protein-Protein Docking

The HDOCK server was used for ab initio template free protein-protein docking in this study (Yan et al., 2017, 2020). For the SARS-CoV-2, SARS-CoV, and MERS-CoV complexes, the main chain of PL^pro^ was defined as the receptor molecule, whereas the ubiquitin chain was the ligand. Protein-protein docking was also performed with PL^pro^ in the presence of compounds that were docked to the naphthalene-inhibitor site in the Schrödinger Suite. This was done for the 6xaa, 4mm3, and 4rf0 crystal structures in order to determine whether the presence of these ligands in the naphthalene-inhibitor region would affect the ability of ubiquitin to bind to PL^pro^.

### Enzymatic Activity Assay

*In vitro* inhibition of the PL^pro^ deubiquitinase activity was measured using a commercially available enzymatic activity assay (BP Bioscience, San Diego, CA, USA). The experiment was performed according to the manufacturer's instructions and all samples were assayed in triplicate. An excitation wavelength of 360 nm was used, and fluorescence was measured at an emission wavelength of 460 nm on the basis of the presence of a ubiquitinated fluoregenic substrate. The non-convalent inhibitor GRL-0617 was provided as an internal positive control and was used at concentration of 100 μM in the assay. We tested the following compounds for potential inhibition of PL^pro^ deubiquitinase activity: hypericin (89%, HWI pharma services GmbH, Germany), cyanidin-3-O-glucoside (reference standard, PhytoLab, Germany), and rutin (>94%), (-)-epicatechin gallate (>98%), (-)-epigallocatechin gallate (>95%), and cefotaxime (European pharmacopeia reference standard) from Sigma-Aldrich (St Luis, MO, USA); 20 mM stock solutions of each compound were stored at −80°C until use. Serial doubling dilutions were performed to yield a final concentration of 3.1 to 200 μM for assaying each compound. Readings (absolute fluorescence values at 1,200 gain), were made using a CLARIOstar^®^ Plus fluorescence microplate reader (BMG Biotech, Oternberg, Germany). Where appropriate the % PL^pro^ deubiquitinase activity was calculated as the ratio of activity in the presence of inhibitor and total activity, and taking into account background readings.

## Results

**Figure 1:**
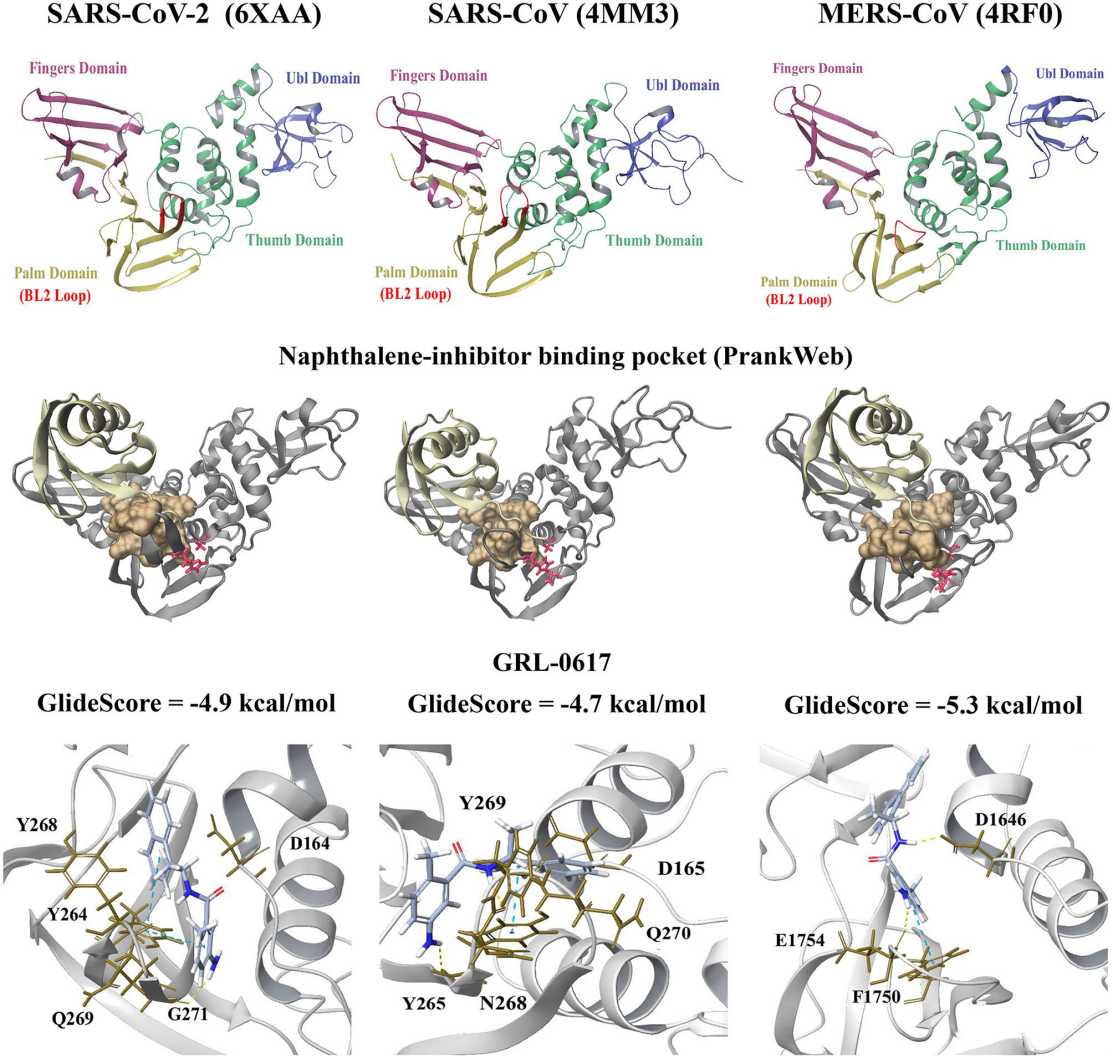
Crystal structures of the SARS-CoV-2 (6xaa), SARS-CoV (4mm3). and MERS-CoV PL^pro^ (4rf0). The fingers, ubiquitin-like (Ubl), thumb and palm domains are labeled. The conserved naphthalene-inhibitor binding pockets that were identified through the PrankWeb server for 6xaa, 4mm3, and 4rf0 are shown. This region is colored ochre and the catalytic triad residues can be seen in red. GRL-0617 (colored violet) was the control and this compound was docked to the naphthalene-inhibitor site of each PL^pro^ structure. The molecular docking results are depicted for 6xaa, 4mm3, and 4rf0. Ubiquitin is colored tan (ribbon representation), while PL^pro^ is colored silver.

Molecular docking was performed to examine the potential inhibitor behavior of dietary compounds and naphthalene inhibitors GRL-0617 and 3k on the SARS-CoV-2 PL^pro^. Like SARS-CoV-2, SARS-CoV, and MERS-CoV are classified as betacoronaviruses and they predominantly affect the respiratory tract (Abdelrahman et al., 2020; Petersen et al., 2020). The PL^pro^ from the novel SARS-CoV-2 was consequently compared to the SARS-CoV and MERS-CoV PL^pro^ structures (Figure 1). In terms of the SARS-CoV and MERS-CoV PL^pro^, the pairwise alignment revealed they had a sequence similarity of 89 and 50% with the SARS-CoV-2 PL^pro^, respectively. The pairwise alignment was used to identify the corresponding residues of the naphthalene-inhibitor binding site for the SARS-CoV and MERS-CoV crystal structures. The three crystal structures (6xaa, 4mm3, and 4rf0) were analyzed using PrankWeb, a binding site prediction tool, which identified the naphthalene-inhibitor binding site as a conserved ligand binding pocket. This region was ranked as pocket 2, pocket 3 and pocket 2 in the SARS-CoV-2, SARS-CoV and MERS-CoV PL^pro^ structures, respectively (Figure 1 and Supplementary Table 1).

**Figure 2:**
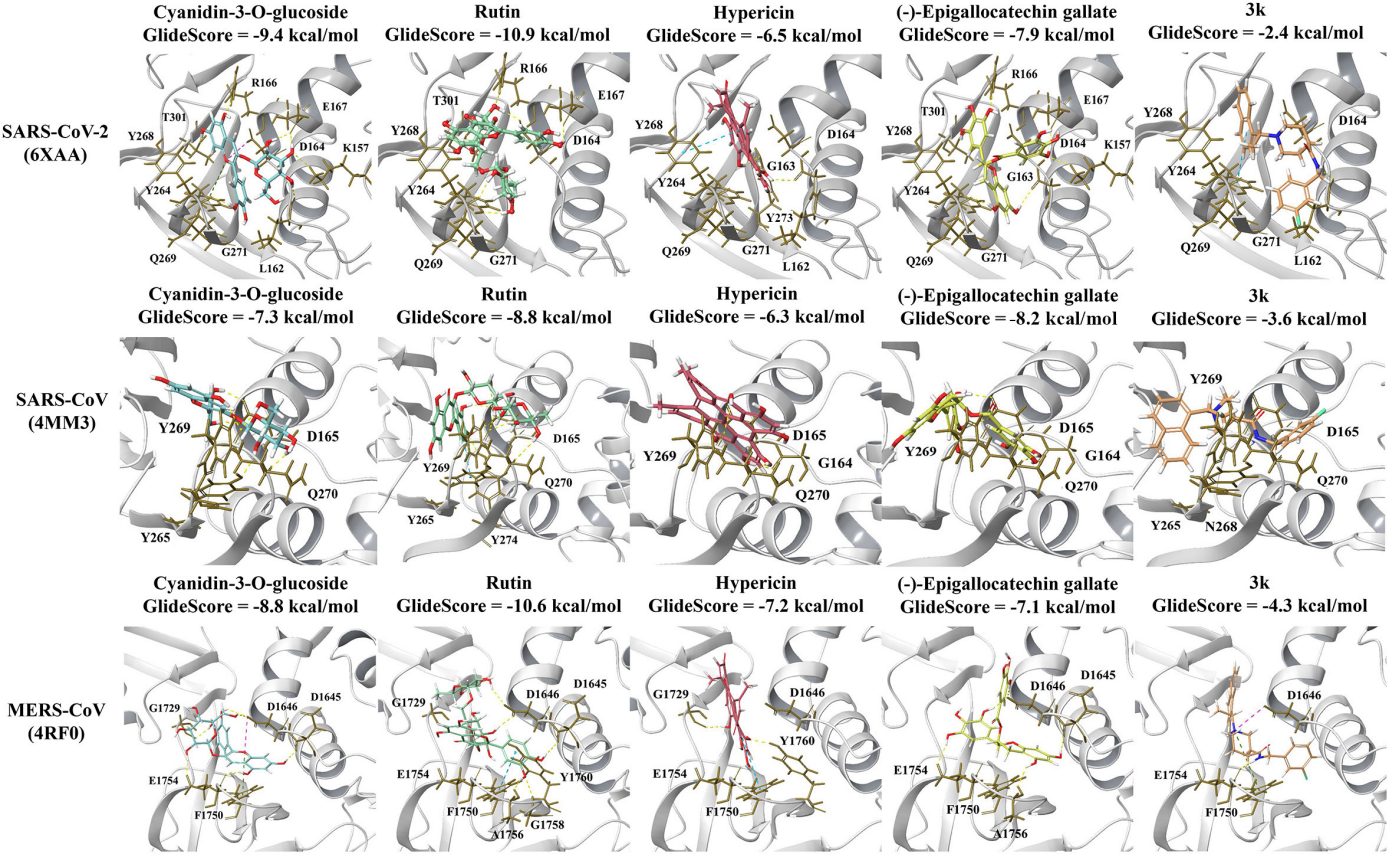
Protein-ligand interactions of the naphthalene-based inhibitor 3k and the dietary compounds. The ligands were docked to the naphthalene-inhibitor site of each protein. The protein-ligand interactions and GlideScores of 3k (orange), cyanidin-3-O-glucoside (blue), rutin (green), hypericin (red), and (-)-epigallocatechin gallate (yellow) are depicted for the SARS-CoV-2 (6xaa), SARS-CoV (4mm3), and MERS-CoV (4rf0) PL^pro^ crystal structures.

GRL-0617 and 3k, as well as the dietary compounds were docked to the naphthalene-inhibitor binding site of the SARS-CoV-2 and SARS-CoV PL^pro^ structures. In the apo 6xaa crystal structure of SARS-CoV-2, the control compound GRL-0617 docked to the naphthalene-inhibitor binding site with a GlideScore of −4.9 kcal/mol (Figure 1). It was predicted to form inter-atomic contacts with G271 (H-bond) and Y264 (π-π interactions). The GlideScore for the naphthalene-based inhibitor 3k was −2.4 kcal/mol and it also formed a π-π interaction with the PL^pro^ residue Y264 (Figure 2). In addition to Y264, a hydrogen bond with L162 was present in the ligand-interaction diagram. The molecular docking results demonstrated that the dietary compounds (-)-epigallocatechin gallate, cyanidin-3-O-glucoside, rutin and hypericin had strong binding affinities (Figure 2). These ligands formed a bond with Y268 (cyanidin-3-O-glucoside: H-bond, (-)-epigallocatechin gallate: H-bond, hypericin: π-π interaction, and rutin: H-bond). The protein residues Y264, T301, D164, R166, E167, K157, L162, G163, and Y273 were also involved in interactions with the ligands (Figure 2).

The GlideScores of the naphthalene-based inhibitors and the dietary compounds for the SARS-CoV and MERS-CoV PL^pro^ structures can be seen in Figures 1, 2. Similar to docking to the same site in the SARS-CoV-2 PL^pro^, the dietary ligands also had stronger binding affinities and formed a greater number of inter-atomic contacts with surrounding residues compared to GRL-0617 and 3k. GRL-0617 formed bonds with Y265 (H-bond and π-π interaction) and N268 (H-bond) in the SARS-CoV PL^pro^, whereas these interactions were absent in docking with 3k (Figures 1, 2). The residues Y265, Y269, Q270, Y274, D165, G164, and N268 were the most prominent amino acids involved in binding with (-)-epigallocatechin gallate, cyanidin-3-O-glucoside, hypericin and rutin (Figure 2). In regards to MERS-CoV, both GRL-0617 and 3k formed inter-atomic contacts with E1754 (GRL-0617: H-bond, 3k: H-bond), F1750 (GRL-0617: π-π interaction, 3k: π-π cation) and D1646 (GRL-0617: H-bond, 3k: salt bridge) (Figures 1, 2). The dietary compounds also interacted with these residues, as well as D1645, G1758, A1756, G1729, and Y1760 (Figure 2).

**Figure 3:**
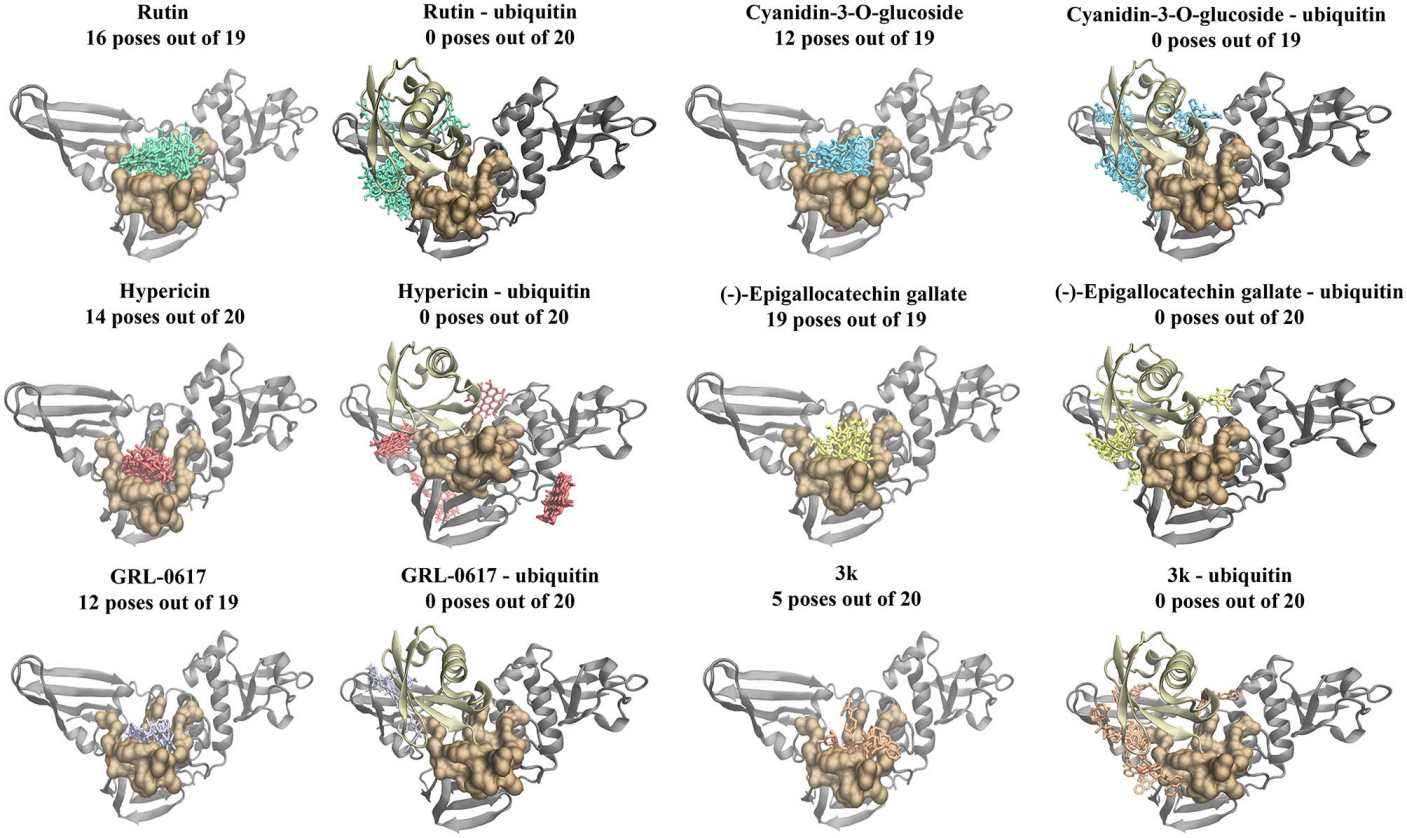
Blind docking results for the SARS-CoV-2 PL^pro^ in the absence and presence of ubiquitin (6xaa). Blind docking was conducted on the apo and ubiquitin-bound crystal structure of the SARS-CoV-2 PL^pro^ using the naphthalene-based inhibitors (GRL-0617: violet, 3k: orange) and the dietary compounds (cyanidin-3-O-glucoside: blue, rutin: green, (-)-epigallocatechin gallate: yellow, hypericin: red). The number of poses that were found to be in the naphthalene-inhibitor binding region (colored ochre) are shown. Ubiquitin is colored tan, while PL^pro^ is colored silver.

Furthermore, blind docking was conducted on the 6xaa (SARS-CoV-2), 4mm3 (SARS-CoV), and 4rf0 (MERS-CoV) crystal structures in the absence of ubiquitin (Figure 3 and Supplementary Figures 1, 2). The blind docking results for the SARS-CoV-2 PL^pro^ can be seen in Figure 3. All six compounds had poses within the naphthalene-inhibitor binding site. For the SARS-CoV-2 and MERS-CoV PL^pro^, all poses generated for (-)-epigallocatechin gallate were predicted to bind to this region (Figure 3 and Supplementary Figure 2). The highest ranking pose that was positioned in the naphthalene-inhibitor binding pocket of the SARS-CoV-2 PL^pro^ was pose 1 for GRL-0617 (−8.0 kcal/mol), pose 4 for 3k (−7.5 kcal/mol), pose 1 for cyanidin-3-O-glucoside (−7.4 kcal/mol), pose 1 for (-)-epigallocatechin gallate (−8.1 kcal/mol), pose 1 for hypericin (−8.5 kcal/mol), and pose 1 for rutin (−7.6 kcal/mol). The binding affinities of the poses that were present in the naphthalene-inhibitor binding region for the 6xaa, 4mm3, and 4rf0 crystal structures can be found in Supplementary Table 2.

Moreover, the six compounds were docked to the 6wuu, 6w9c, 6wx4, and 7jrn crystal structures of the SARS-CoV-2 PL^pro^ (Supplementary Figures 3–6). With the exception of the 7jrn PL^pro^ structure, all of the dietary compounds had stronger GlideScores than the naphthalene-based inhibitors. Hypericin, rutin and (-)-epigallocatechin gallate were predicted to bind more strongly than GRL-0617 to the 7jrn PL^pro^ structure, while cyanidin-3-O-glucoside had a similar GlideScore to the control compound. In the 6w9c, 6wuu, and 7jrn SARS-CoV-2 PLpro structures, 3k formed bonds with Y264 (6wuu: H-bond, 6w9c: H-bond, and salt bridge, 7jrn: salt bridge). In 7jrn, 3k formed a hydrogen bond with Y268. The intermolecular bonds that GRL-0617 formed with the protein residues varied amongst the crystal structures. GRL-0617 formed two hydrogen bonds with G266 and N267 in 6w9c, whereas a hydrogen bond was present with D164 in 6wuu. In 6wx4, there were two π-π interactions with Y264 and hydrogen bonds with G271 and Y268. There were π-π interactions with Y268 in 7jrn and a hydrogen bond with Q269.

Compared to GRL-0617 and 3k, the ligands rutin, cyanidin-3-O-glucoside and (-)-epigallocatechin gallate formed a greater number of inter-atomic contacts with the protein residues (Supplementary Figures 3–6). In 6w9c, the dietary compounds formed hydrogen bonds with D164. Hydrogen bonds were also present with D164 for (-)-epigallocatechin gallate and rutin in the 6wuu and 7jrn structures. The amino acid Y268 was also frequently involved in intermolecular bonds with the dietary compounds. This included (-)-epigallocatechin gallate (6w9c: H-bond), cyanidin-3-O-glucoside (6wuu: H-bond, 7jrn: π-π interaction), rutin (6wuu: H-bond and 6wx4: H-bond), and hypericin (6wx4: π-π interaction and 7jrn: π-π interactions). In the 7jrn PL^pro^, hypericin also formed a hydrogen bond with Q269 and this was similar to the control compound. The blind docking results for the 6xaa, 6w9c, 6wx4, and 7jrn SARS-CoV-2 PL^pro^ crystal structures can be found in Supplementary Table 3.

**Figure 4:**
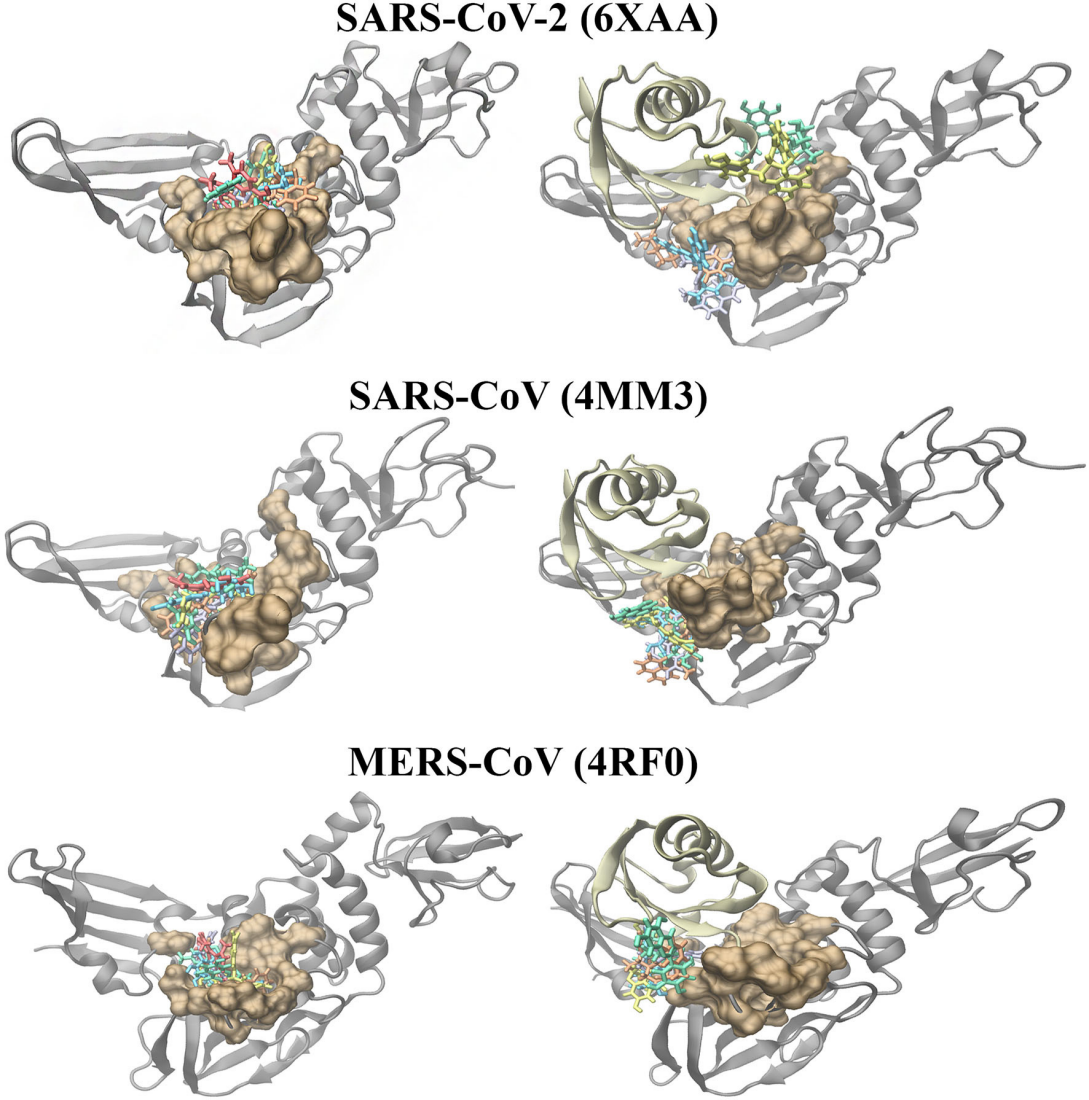
Molecular docking results of the naphthalene-based inhibitors and the dietary compounds to the SARS-CoV-2 (6xaa), SARS-CoV (4mm3), and MERS-CoV (4rf0) PL^pro^ in the absence and presence of ubiquitin. The compounds were docked to the naphthalene-inhibitor binding region (colored ochre) of the apo and ubiquitin-bound PL^pro^ crystal structures. Ubiquitin is colored tan, while PL^pro^ is colored silver. GRL-0617 is colored violet, 3k is colored orange, cyanidin-3-O-glucoside is colored blue, (-)-epigallocatechin gallate is colored yellow, rutin is colored green and hypericin is colored red.

Molecular docking was also performed on the SARS-CoV-2, SARS-CoV and MERS-CoV PL^pro^ structures with the ubiquitin chain present (Figure 4). When GRL-0617, 3k, (-)-epigallocatechin gallate, rutin, cyanidin-3-O-glucoside, and hypericin were docked to PL^pro^, it was evident that they were binding distant from the naphthalene-inhibitor binding site. The position and orientation of these ligands, as well as their GlideScores, differed to when ubiquitin was absent (Figure 4 and Supplementary Table 4). GRL-0617 in the SARS-CoV-2 PL^pro^, for example, was found to form hydrogen bonds with P248 and G266. The control compound was no longer binding to the pocket that the C-terminal chain of ubiquitin extends into. Interestingly, hypericin was not able to bind in the presence of ubiquitin. Similarly, the blind docking results for PL^pro^ in complex with ubiquitin revealed that the compounds that originally had poses within the naphthalene-inhibitor binding site were displaced from this region.

**Figure 5:**
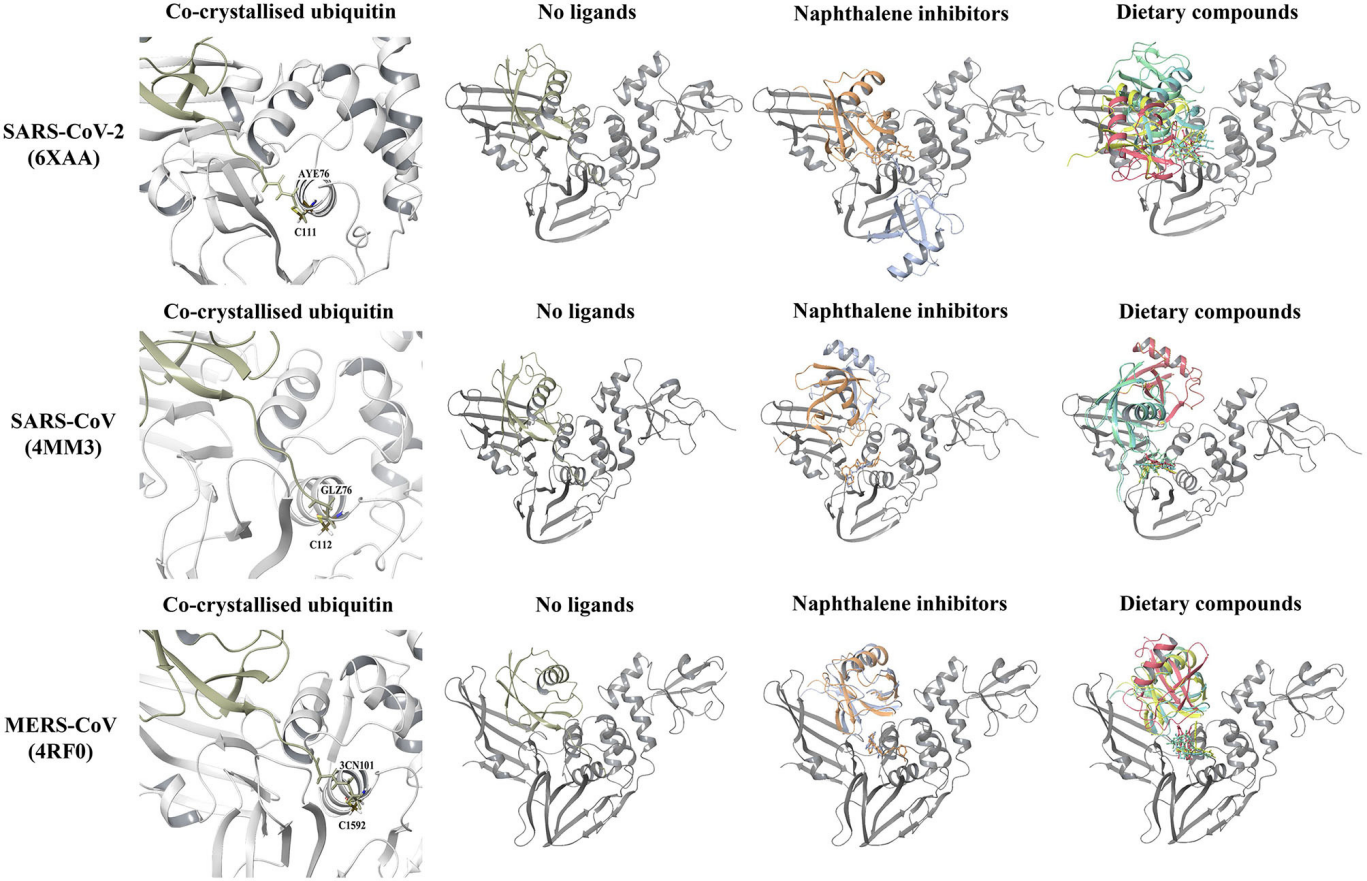
Protein-protein docking results of the SARS-CoV-2 (6xaa), SARS-CoV (4mm3), and MERS-CoV (4rf0) PL^pro^-ubiquitin complexes. The covalent bond that is formed between the C-terminal chain of the crystallized ubiquitin and the catalytic cysteine residue of the proteins can be seen. The HDOCK server was used to dock ubiquitin to the apo PL^pro^ and to PL^pro^ with ligands bound to the naphthalene-inhibitor region. Ubiquitin is colored tan, while PL^pro^ is colored silver. GRL-0617 is colored violet, 3k is colored orange, cyanidin-3-O-glucoside is colored blue, (-)-epigallocatechin gallate is colored yellow, rutin is colored green and hypericin is colored red.

The PL^pro^-ubiquitin complexes were investigated further using the HDOCK server for protein-protein docking (Figure 5). The ubiquitin chain from each complex was isolated and was re-docked to the main PL^pro^ structure. There was a clear overlap in the position between the top ranked model of the docked ubiquitin and the crystallographic ubiquitin. The root-mean square deviation (RMSD) values that were generated from protein-protein docking were 0.4, 0.6, and 0.5 Å for the SARS-CoV-2, SARS-CoV, and MERS-CoV complexes, respectively, and importantly, the C-terminal tail of ubiquitin extended into the catalytic and naphthalene binding region of PL^pro^. The C-terminus of ubiquitin in the SARS-CoV-2, SARS-CoV and MERS-CoV PL^pro^ consists of residues R72, L73, R74, and G75. The C-terminal residue of ubiquitin is AYE76, GLZ76, and 3CN101 in the SARS-CoV-2, SARS-CoV and MERS-CoV structures, respectively. These three residues form a covalent bond with the catalytic cysteine residues and this is critical for deubiquitinase activity (C111 in SARS-CoV-2: 1.67 Å, C112 in SARS-CoV: 1.72 Å and C1592 in MERS-CoV: 1.45 Å). The 1.67, 1.72, and 1.45 Å correspond to the distances between the sulfur atom of the catalytic cysteine residue and the C-terminal ubiquitin residue in the crystal structures. Although non-covalent protein-protein docking was performed in the present study, the distance between AYE76 and C111 in SARS-CoV-2 was 1.94 Å. The distance between GLZ76 and residue C112 in SARS-CoV was 2.05 Å. Likewise, the distance between 3CN101 and C1592 in MERS-CoV was 1.48 Å.

In order to examine the ability of ubiquitin to bind to PL^pro^ when compounds are present in the naphthalene-inhibitor binding site, the ligands that were docked using the Schrödinger Suite were retained in the protein structures (Figure 5). When GRL-0617, 3k, (-)-epigallocatechin gallate, rutin, cyanidin-3-O-glucoside, and hypericin were bound to this region, dramatic differences were observed in the binding mode of ubiquitin. The C-terminal chain of ubiquitin was no longer extending into the pocket that is located just above the catalytic triad. There were consequently changes in the position and orientation of ubiquitin for each ligand. When comparing the docked ubiquitin chain to the ubiquitin present in the original crystal structure, the RMSD values and docking scores were different (Supplementary Table 5). This was apparent for the SARS-CoV-2, SARS-CoV and MERS-CoV complexes.

### Hypericin, Rutin, and Cyanidin-3-O-Glucoside Inhibit PL^pro^ Deubiquitinase Activity in a Concentration-Dependent Manner

**Figure 6:**
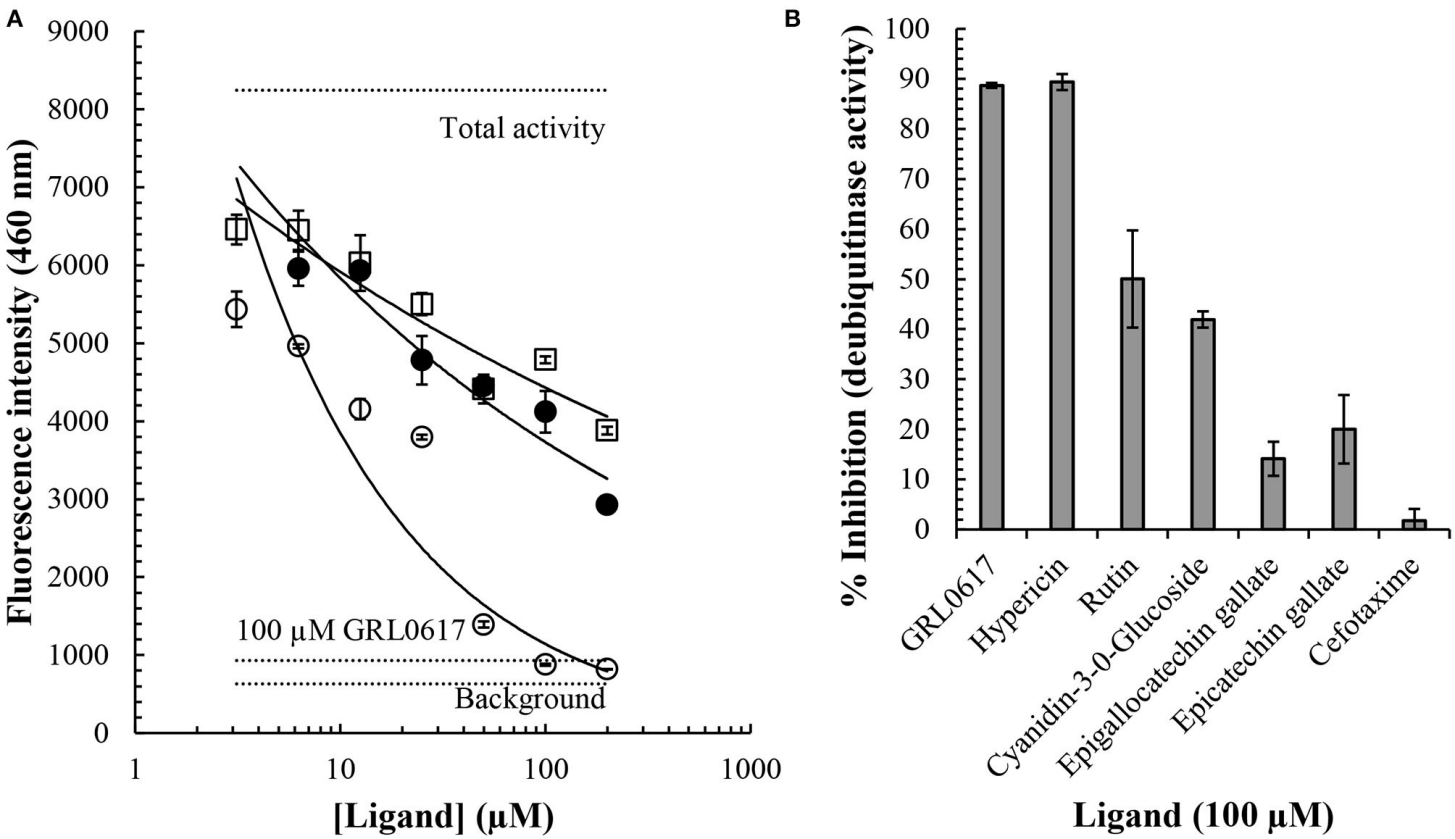
Inhibition of the SARS-CoV-2 PL^pro^ deubiquitinase activity by small molecules. A commercially available papain-like protease (SARS-CoV-2) assay kit measuring deubiquitinase activity was used to investigate the effects of small molecules *in vitro* (BPS Bioscience). The presence of an ubiquinated fluoregenic substrate was measured at an emission wavelength of 460 nm. Cyanidin-3-O-glucoside (open squares), rutin (closed circles), and hypericin (open circles), resulted in concentration-dependent inhibition of PL^pro^ deubiquitinase activity **(A)**. Concentration ranges between 3 and 200 μM were investigated and average values ± SEM are shown. Horizontal dotted lines indicate the total activity (*n* = 3), background (*n* = 3), and inhibition by the internal positive control, GRL-0617 (*n* = 3). The % inhibition of PL^pro^ deubiquitinase activity by a series of compounds at 100 μM is shown **(B)**. Data indicates average values ± SEM from triplicate samples.

Inhibition of PL^pro^ deubiquitinase activity by small molecules *in vitro* was investigated using an enzymatic assay. Overall, the findings indicate that hypericin, rutin, and cyanidin-3-O-glucoside resulted in a concentration-dependent inhibition of PL^pro^ deubiquitinase activity, with hypericin clearly highlighting the most potent inhibition of the test ligands (Figure 6A). At the higher concentrations (>50 μM) hypericin inhibited PL^pro^ deubiquitinase activity to a level akin to the internal positive control (GRL-0617), which was used at 100 μM in the assay. Indeed, calculation of the percentage inhibition of PL^pro^ deubiquitinase activity at 100 μM for each ligand highlighted the equivalence of GRL-0617 and hypericin in the assay (inhibition of activity by ~90% by both compounds, Figure 6B). The findings indicate that at 100 μM both rutin (~50% inhibition) and cyanidin-3-O-glucoside (~42% inhibition), can also be considered as potentially useful inhibitors of PL^pro^ deubiquitinase activity. The epicatechins (epigallocatechin gallate, ~14% and epicatechin gallate, ~20%), and cefotaxime (~2%), yielded more modest effects.

## Discussion

The innate immune system is the first line of defense against foreign pathogens and various cellular and molecular components are involved in this process (Liu et al., 2016; Huang et al., 2019). Post-translational modifications are also important regulators of immunity and this includes ubiquitination (Liu et al., 2016). Ubiquitin is a 76-amino acid polypeptide that can covalently interact with target proteins and ubiquitin itself can undergo ubiquitination at certain residues (Ciechanover et al., 2000; Pickart, 2001; Jiang and Chen, 2011). This results in the formation of lysine-linked polyubiquitin chains or linear polyubiquitin chains (Heaton et al., 2015). It is well-known that lysine 48 (K48)-linked polyubiquitylation promotes the proteasomal degradation of target proteins (Ciechanover et al., 2000). Conversely, lysine 63 (K63)-linked polyubiquitylation has been implicated in cellular processes such as the DNA damage response, inflammation and endocytosis (Panier and Durocher, 2009; Erpapazoglou et al., 2014; Zhou Z. et al., 2020). It is also important to note that other types of polyubiquitin chain linkages are being explored and that ubiquitin modifications can lead to different cellular outcomes (Komander and Rape, 2012; Ohtake et al., 2018).

Moreover, human deubiquitinases (DUBs) are enzymes that remove ubiquitin modifications and they contribute to homeostasis (Li et al., 2016). Viruses are dependent on host cells for their survival and in order to complete their life cycle, they have developed strategies to evade the antiviral immune response (Nelemans and Kikkert, 2019). Interestingly, several viral proteins have been found to possess deubiquitinating activity and they can be used to antagonize or modulate the antiviral immune signaling pathway (Kumari and Kumar, 2018).

The deubiquitinating activity of the SARS-CoV-2 PL^pro^ was the main focus of this study and the crystal structure of PL^pro^ in complex with ubiquitin propargylamide was utilized (Klemm et al., 2020). In addition to this, four other crystal structures of SARS-CoV-2 that were available on the RCSB PDB were evaluated. The crystal structures of the SARS-CoV PL^pro^-ubiquitin aldehyde and MERS-CoV PL^pro^-ubiquitin complexes were used for comparison (Bailey-Elkin et al., 2014; Ratia et al., 2014). Molecular docking allowed for the binding properties of compounds to the known target site of naphthalene-based inhibitors to be predicted and examined.

GRL-0617 was the control and this has previously been found to potently inhibit the SARS-CoV and SARS-CoV-2 PL^pro^ in a non-covalent manner (Ratia et al., 2008; Freitas et al., 2020; Shin et al., 2020). The GRL-0617 inhibitor occupies the S3 and S4 pockets of the SARS-CoV and SARS-CoV-2 PL^pro^ (Ratia et al., 2008; Gao et al., in press). Based on the molecular docking results from the current study, GRL-0617 was predominantly surrounded by hydrophobic residues in the SARS-CoV and SARS-CoV-2 crystal structures (Figure 1 and Supplementary Figures 3–6) (Ratia et al., 2008). This ligand was predicted to form inter-atomic contacts with the protein residues and this included D164 in the 6wuu SARS-CoV-2 structure, as well as Q269 in the 7jrn SARS-CoV-2 structure. In the crystal structure determined by Gao et al., GRL-0617, which was the co-crystallized ligand, was found to form hydrogen bonds with these critical residues (Gao et al., in press). In saying this, D164 and Q269 were found to surround GRL-0617 in the 6xaa, 6w9c, and 6wx4 structures of the SARS-CoV-2 PL^pro^.

GRL-0617 also interacted with Y268 in the SARS-CoV-2 PL^pro^ and inter-atomic contacts were present with this residue in some structures. Interestingly, the naphthalene-based inhibitor 3k was found to consistently form inter-atomic contacts with Y264 in all of the SARS-CoV-2 crystal structures (Figure 2 and Supplementary Figures 3–6) (Bosken et al., 2020). Intermolecular bonds were also formed between 3k and D164 in three of the SARS-CoV-2 papain-like proteases, as well as Y268 in the 6wx4 structure (Bosken et al., 2020). Bosken et al. have identified these residues as playing an important role in the binding mode of 3k (Bosken et al., 2020).

The differences observed in the intermolecular bonds may be due to the conformations of the fingers domain and BL2 loop in the PL^pro^ crystal structures (Figure 1) (Báez-Santos et al., 2015). In the SARS-CoV-2 and SARS-CoV PL^pro^ structures, the BL2 loop corresponds to residues 267-272 (Lee et al., 2015; Gao et al., in press). In the MERS-CoV PL^pro^ structure used in this study, the BL2 loop is comprised of residues 1,752–1,758 (Bailey-Elkin et al., 2014; Lee et al., 2015). The structural significance of the BL2 loop (blocking loop) has been discussed in a number of papers and its flexibility has been highlighted (Báez-Santos et al., 2015; Bosken et al., 2020; Klemm et al., 2020). Conformational changes have been observed in the BL2 loop and “open” or “closed” conformations have been reported in the literature (Báez-Santos et al., 2015). In regards to MERS-CoV, there are significant structural differences in the BL2 loop and it has been suggested that this affects inhibitor recognition specificity (Lee et al., 2015).

GRL-0617 is ineffective against MERS-CoV and in the study by Shin et al., it was discussed that this may be due to the presence of a threonine residue instead of tyrosine at a conserved position (Lee et al., 2015; Shin et al., 2020). In the SARS-CoV-2 and SARS-CoV PL^pro^ sequences, the corresponding residues are Y268 and Y269, respectively (Shin et al., 2020). Y268 is required for the inhibitory effect of GRL-0617 and Shin et al. demonstrated that mutating this residue strongly reduces its potency (Shin et al., 2020). While GRL-0617 and 3k were predicted to bind to the MERS-CoV PL^pro^ (4rf0) in this study, further docking to additional crystal structures may be required for comparison (Figures 1, 2). The naphthalene-based inhibitors were surrounded by the residues D165, Y269, and Q270 in the SARS-CoV PL^pro^. Ratia et al. and Báez-Santos et al., have also described the importance of these residues in the mechanisms of action of these ligands (Ratia et al., 2008; Báez-Santos et al., 2014).

Most notably, the dietary compounds (-)-epigallocatechin gallate, hypericin, rutin and cyanidin-3-O-glucoside were predicted to bind more strongly to the naphthalene-inhibitor site of the SARS-CoV-2, SARS-CoV, and MERS-CoV papain-like proteases than the known inhibitors (Figure 2 and Supplementary Figures 3–6). They also formed multiple interactions with the key protein residues compared to GRL-0617 and 3k. Similarly, the blind docking results on the main PL^pro^ chains showed that these natural ligands had multiple poses within this region (Figure 3 and Supplementary Figures 1, 2). (-)-Epigallocatechin gallate, rutin and cyanidin-3-O-glucoside are flavonoids, a biologically active class of the phenolic compounds (Bonvino et al., 2018). In a recent literature review conducted by Verma et al., the flavonoids were found to be the largest class of compounds with potential activity against coronaviruses (Verma et al., 2020).

In 2005, Li et al. published a study about the antiviral activities of natural compounds against SARS-CoV (Li et al., 2005). Lycorine was identified as a potent antiviral compound and potentially a candidate for the development of new medicines (Li et al., 2005). Natural compounds have been screened for their ability to target SARS-CoV-2 proteins. This includes extracts of medicinal herbs and several studies have focused on their inhibitory effects on key proteins, such as the spike glycoprotein and the main protease (M^pro^) (Mani et al., 2020; Pitsillou et al., 2020; Russo et al., 2020; Smith and Smith, 2020). In a recent paper by Alamri et al. a structured-based computational approach was utilized to identify compounds that may act as pan-PL^pro^ inhibitors and could be developed further as antiviral agents (Alamri et al., in press). Given the current situation, *in silico* methods have made it possible for large libraries of existing approved compounds to be screened in a relatively fast manner (Ojha et al., 2020). The structures of the hits identified from these computational studies could be optimized as part of the drug discovery process (Ojha et al., 2020). In addition to synthetic pharmacological compounds, herbal constituents can be screened in the same manner and this method has been described in many papers (Bhowmik et al., 2020; Chikhale et al., 2020; Ghosh et al., 2020; Gupta et al., 2020; Jena et al., 2020; Krupanidhi et al., 2020; Muhseen et al., 2020; Sinha et al., 2020; Subbaiyan et al., 2020). A number of studies that can be found on the World Health Organization's International Clinical Trials Registry Platform also involve plant-based compounds, particularly flavonoids.

Ratia et al. determined the crystal structure of SARS-CoV in complex with ubiquitin aldehyde and they described how this polypeptide interacts with the palm and fingers regions of PL^pro^ (Ratia et al., 2014). They emphasized that a significant amount of the binding energy of ubiquitin can be attributed to its C-terminal residues (R72-G76) and that this portion of ubiquitin forms an extensive number of intermolecular hydrogen bonds with PL^pro^ (Ratia et al., 2014). The results from their study also indicated that the SARS-CoV PL^pro^ had a preference for K48-linked ubiquitin and ISG15, over K63-polyubiquitin chains and mono-ubiquitin (Ratia et al., 2014). Most notably, two recognition sites on the surface of PL^pro^ were characterized and were defined as either SUb1 or SUb2 (Ratia et al., 2014).

In the SARS-CoV-2 PL^pro^ complex, ubiquitin propargylamide sits on the same subdomains as the SARS-CoV structure (palm and fingers regions), and the C-terminus extends into the active site (Klemm et al., 2020). Like SARS-CoV, the SARS-CoV-2 PL^pro^ was also found to have a second ubiquitin binding site (SUb2) that is important for the binding of polyubiquitin (K48-diubiquitin) and ISG15 (Klemm et al., 2020). The MERS-CoV (space group P6_5_22) PL^pro^-ubiquitin complex was solved by Bailey-Elkin et al. and in their paper, they refer to this structure as the closed conformation since the fingers domain is shifted toward the ubiquitin (Bailey-Elkin et al., 2014).

When ubiquitin was present in the SARS-CoV-2, SARS-CoV and MERS-CoV PL^pro^ structures, the results from Schrödinger and blind docking showed that compounds were displaced from the naphthalene-inhibitor binding pocket (Figures 3, 4 and Supplementary Figures 1, 2). Compared to the apo PL^pro^, hypericin was unable to produce molecular docking poses for the PL^pro^-ubiquitin complexes. Likewise, the protein-protein docking results with the ligands already bound to this region in PL^pro^ revealed that ubiquitin was binding in different conformations and that the position of the C-terminus was altered (Figure 5). This suggests that the dietary compounds may be able to interfere with the deubiquitinase activity of PL^pro^ and in terms of *in silico* methods, this can be evaluated further using molecular dynamics (MD) simulations.

A commercially available PL^pro^ enzymatic assay was used to measure deubiquitinase activity using GRL-0617 as an internal positive control; in this specific assay GRL-0617 has been shown to have an IC_50_ value of 1.7 μM for inhibition of PL^pro^ deubiquitinase activity (BP Bioscience). Overall, our findings indicated inhibition with an order of potency of GRL-0617 and hypericin > rutin and cyanidin-3-O-glucoside > epigallocatechin gallate and epicatechin gallate >> cefotaxime. GRL-0617 and hypericin > rutin > cyanidin-3-O-glucoside and epicatechin gallate > cefotaxime and epigallocatechin gallate (Figure 6). The potent inhibition of PL^pro^ deubiquitinase activity by hypericin which, at higher concentrations, was analogous to GRL-0617, is particularly encouraging. Hypericin is an anthraquinone derivative that can be found in the flowering plant *Hypericum perforatum*, which is also commonly known as St. John's Wort (Napoli et al., 2018). It has been identified as a lead compound for the SARS-CoV-2 spike protein and its antiviral properties have been the subject of numerous papers in the past (Jacobson et al., 2001; Shih et al., 2018; Chen et al., 2019). In addition to its antiviral effects, St. John's Wort is also being investigated for its antidepressant properties and synthetic hypericin (SGX301) has gained attention for its use as a photodynamic agent in the treatment of cutaneous T-cell lymphoma (Rook et al., 2010; Montoya et al., 2015; Apaydin et al., 2016).

While specific compounds were selected for use in this study, it would be important to expand this in the future to incorporate a greater number of phytochemicals that are present in various plant extracts. Network pharmacology is also being increasingly used in drug discovery and this systematic approach can assist with identifying potential protein targets and lead compounds, as well as understanding their mechanisms of action (Zhang et al., 2019; Pan H. D. et al., 2020). Nonetheless, the antiviral, antioxidant and anti-inflammatory properties of the compounds used in this study had been previously reported in the literature and were consequently suitable candidates. Molecular docking was used for virtual screening and although the scoring functions produced from docking aren't absolute binding energies, it allowed for predictions to be made about the protein-ligand interactions. Docking was performed using the Glide (XP) protocol of the Schrödinger Suite and in a study conducted by Wang et al., this was found to have a 90% success rate in identifying the correct binding poses of ligands (Wang et al., 2016). In this study, the inhibitory activities of the compounds were subsequently measured using an enzymatic activity assay. *In silico* tools are currently being utilized for the early stages of the drug discovery pipeline however, it is important to note that the pipeline involves multiple steps and is a time consuming process (Agostino et al., 2019). Potential drugs must be explored further in pre-clinical trials using a combination of techniques and clinical trials (Agostino et al., 2019).

## Conclusion

Overall, on the basis of our *in silico* and *in vitro* evaluations, hypericin, rutin, and cyanidin-3-O-glucoside can be considered potential lead compounds. In particular, further clarification of the molecular mechanisms and antiviral properties of hypericin, which displayed high potency in the *in vitro* assay and favorable binding properties in the *in silico* studies, is warranted.

## Data Availability Statement

The original contributions presented in the study are included in the article/Supplementary Material, further inquiries can be directed to the corresponding author/s.

## Author Contributions

TK and AH conceptualized the aims and methodology and were involved in supervision. TK was involved in the production of the first draft of the manuscript. EP performed data analysis, data curation, and was involved in production of the first draft of the manuscript. JL was involved in data analysis and curation and was involved in production of the first draft of the manuscript. KV performed formal data analysis and was involved in data curation. KL performed formal data analysis and validation. All authors contributed to editing and reviewing the manuscript.

## Conflict of Interest

Epigenomic Medicine Program (TCK) is supported financially by McCord Research (Iowa, USA), which has a financial interest in dietary compounds described in this work. However, there is no conflict of interest with respect to the inhibition of the SARS-CoV-2 papain-like protease. The remaining authors declare that the research was conducted in the absence of any commercial or financial relationships that could be construed as a potential conflict of interest.

## Supplementary Material

Supplementary Figure 1 Blind docking of the naphthalene-based inhibitors and dietary compounds to the SARS-CoV PL^pro^.

Supplementary Figure 2 Blind docking of the naphthalene-based inhibitors and dietary compounds to the MERS-CoV PL^pro^.

Supplementary Figure 3 Molecular docking results of the naphthalene-based inhibitors and dietary compounds for the SARS-CoV-2 PL^pro^ structure 6w9c.

Supplementary Figure 4 Molecular docking results of the naphthalene-based inhibitors and dietary compounds for the SARS-CoV-2 PL^pro^ structure 6wuu.

Supplementary Figure 5 Molecular docking results of the naphthalene-based inhibitors and dietary compounds for the SARS-CoV-2 PL^pro^ structure 6wx4.

Supplementary Figure 6 Molecular docking results of the naphthalene-based inhibitors and dietary compounds for the SARS-CoV-2 PL^pro^ structure 7jrn.

Supplementary Table 1 PrankWeb conservation analysis results for the SARS-CoV-2 (6xaa), SARS-CoV (4mm3), and MERS-CoV PL^pro^ (4rf0).

Supplementary Table 2 Blind docking of the naphthalene-based inhibitors and dietary compounds to the SARS-CoV-2 (6xaa), SARS-CoV (4mm3), and MERS-CoV (4rf0) PL^pro^ structures.

Supplementary Table 3 Blind docking of the naphthalene-based inhibitors and dietary compounds to the SARS-CoV-2 6w9c, 6wuu, 6wx4, and 7jrn PL^pro^ crystal structures. No poses were found in the naphthalene inhibitor site for GRL-0617 and hypericin in the 6wuu structure.

Supplementary Table 4 The GlideScores (kcal/mol) of the naphthalene-based inhibitors and dietary compounds for the SARS-CoV-2, SARS-CoV, and MERS-CoV crystal structures with ubiquitin are listed.

Supplementary Table 5 The docking scores and RMSD values for the top-ranked PL^pro^-ubiquitin complexes from the HDOCK server are provided.
