# Supplementary figures and images for: Identification of Small Molecule Inhibitors of the Deubiquitinating Activity of the SARS-CoV-2 Papain-Like Protease: in silico Molecular Docking Studies and in vitro Enzymatic Activity Assay
Source: Front Chem. 2020 Dec 8;8:623971. doi: 10.3389/fchem.2020.623971 (PMC7753156; doi:10.3389/fchem.2020.623971)

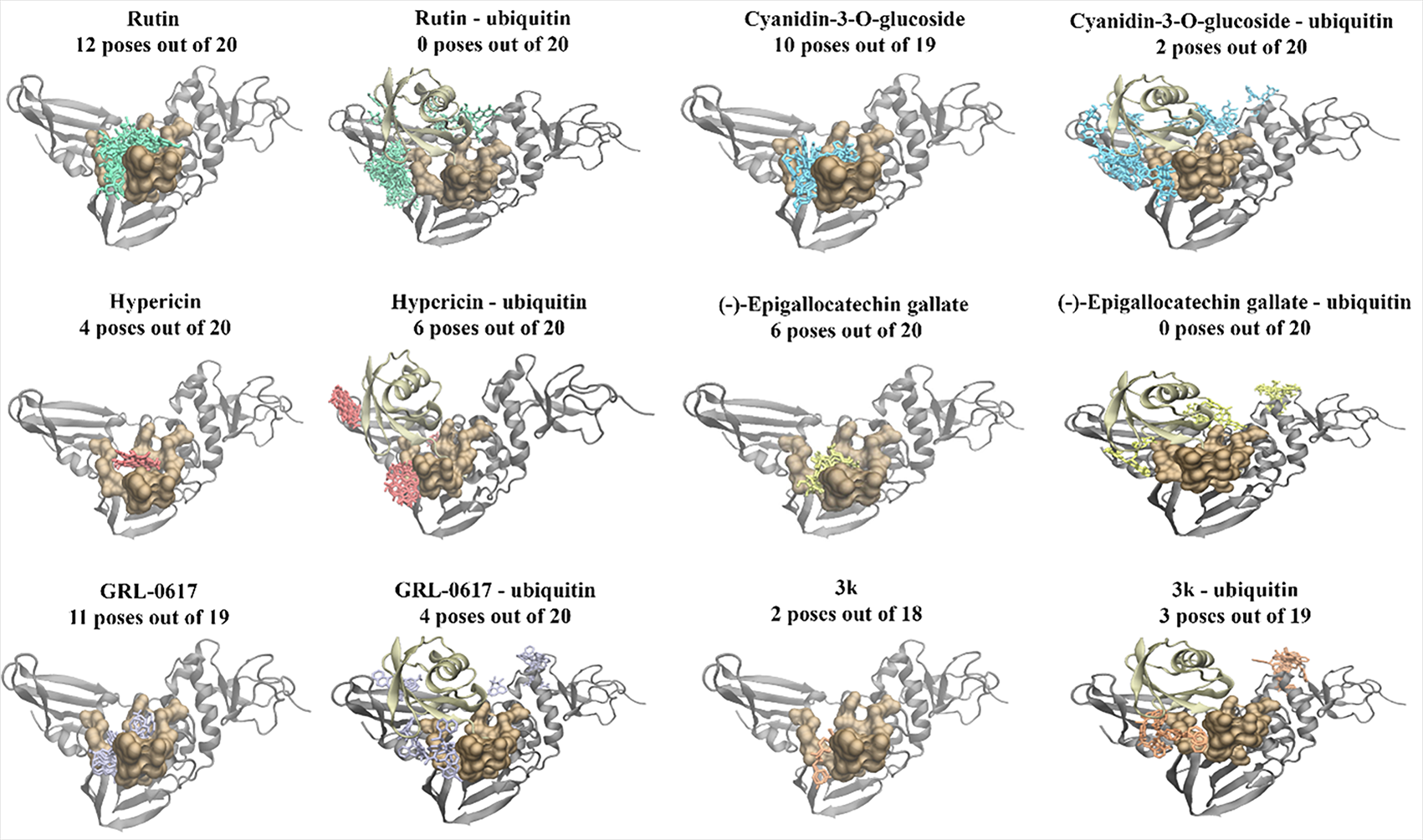

Supplement: Supplementary Figure 1 — Blind docking of the naphthalene-based inhibitors and dietary compounds to the SARS-CoV PLpro. [file Image_1.TIF]

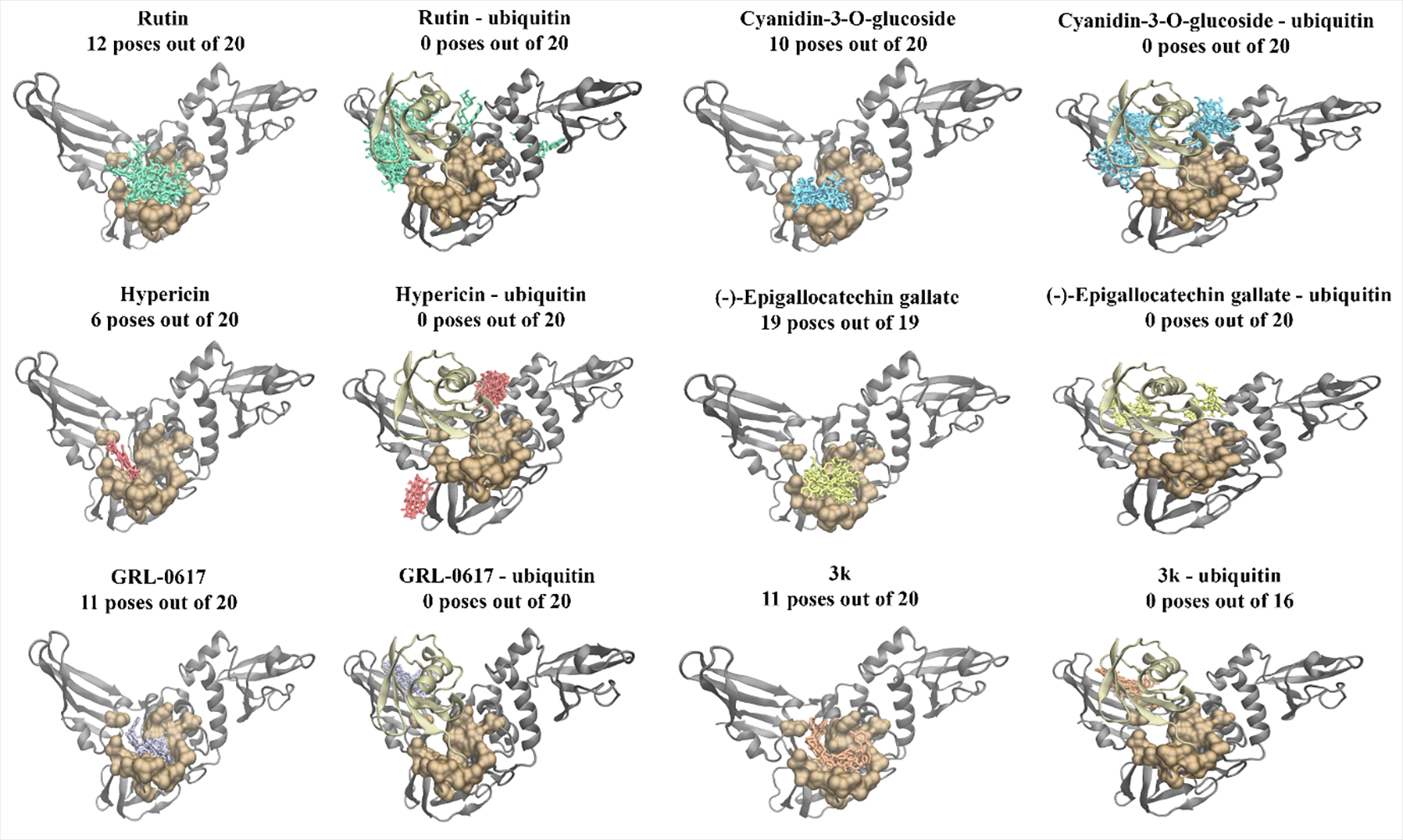

Supplement: Supplementary Figure 2 — Blind docking of the naphthalene-based inhibitors and dietary compounds to the MERS-CoV PLpro. [file Image_2.TIF]

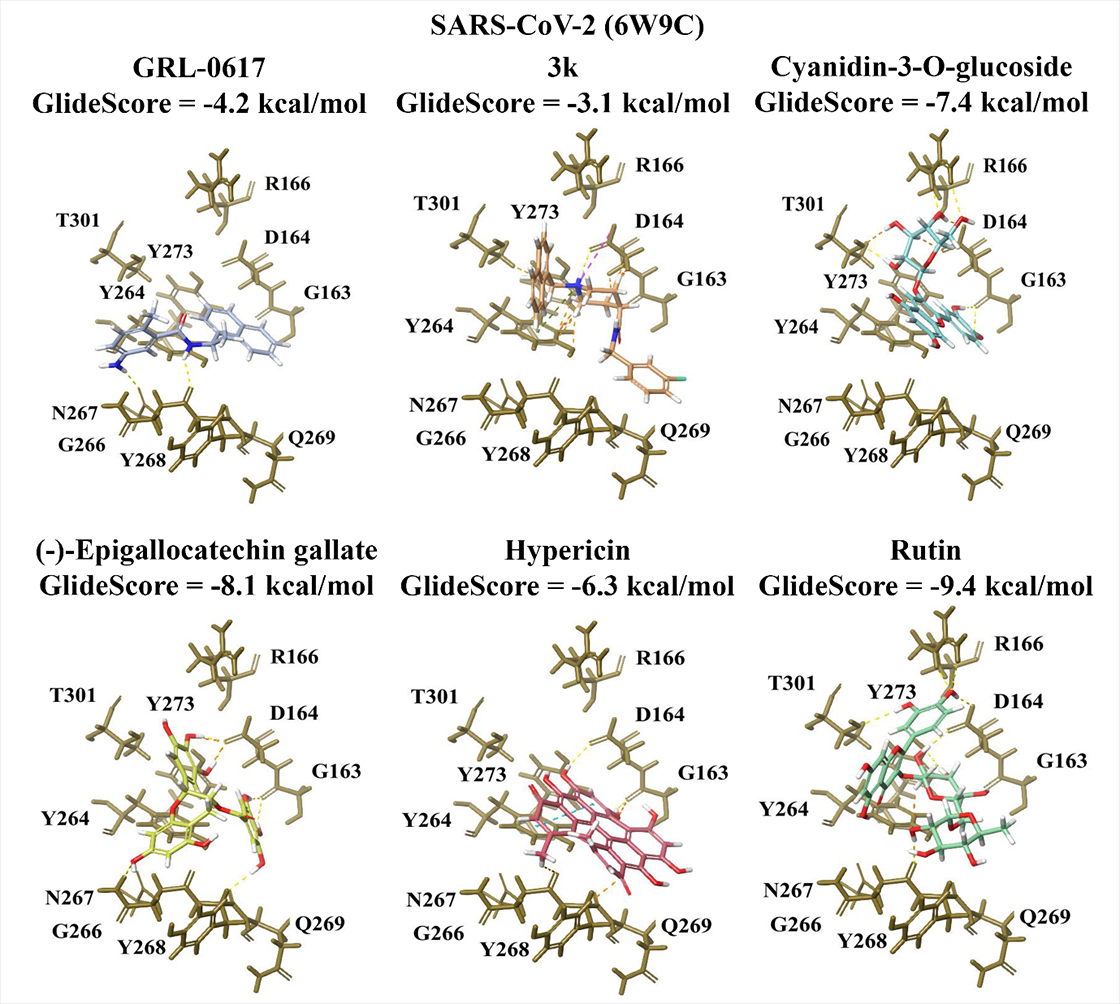

Supplement: Supplementary Figure 3 — Molecular docking results of the naphthalene-based inhibitors and dietary compounds for the SARS-CoV-2 PLpro structure 6w9c. [file Image_3.TIF]

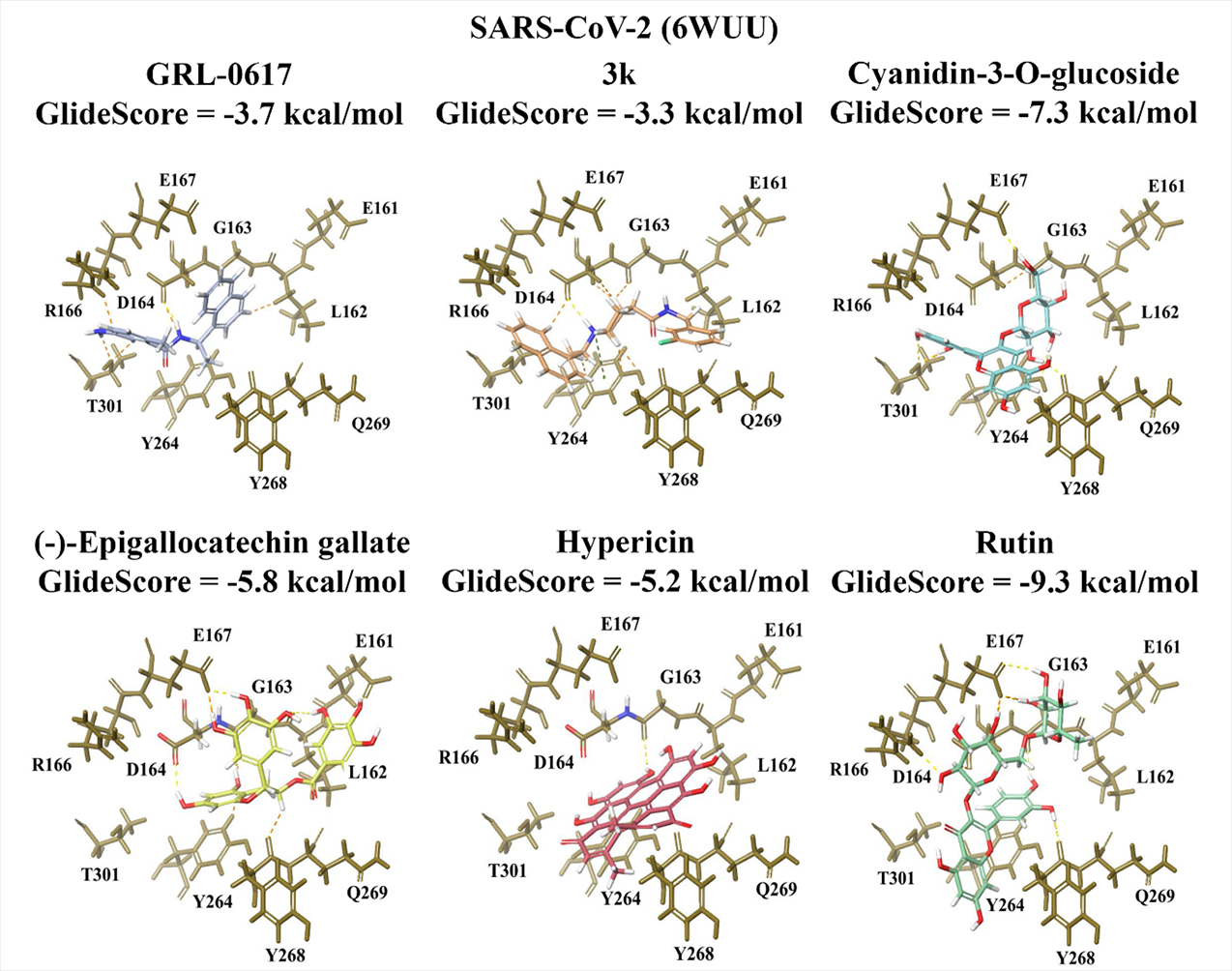

Supplement: Supplementary Figure 4 — Molecular docking results of the naphthalene-based inhibitors and dietary compounds for the SARS-CoV-2 PLpro structure 6wuu. [file Image_4.TIF]

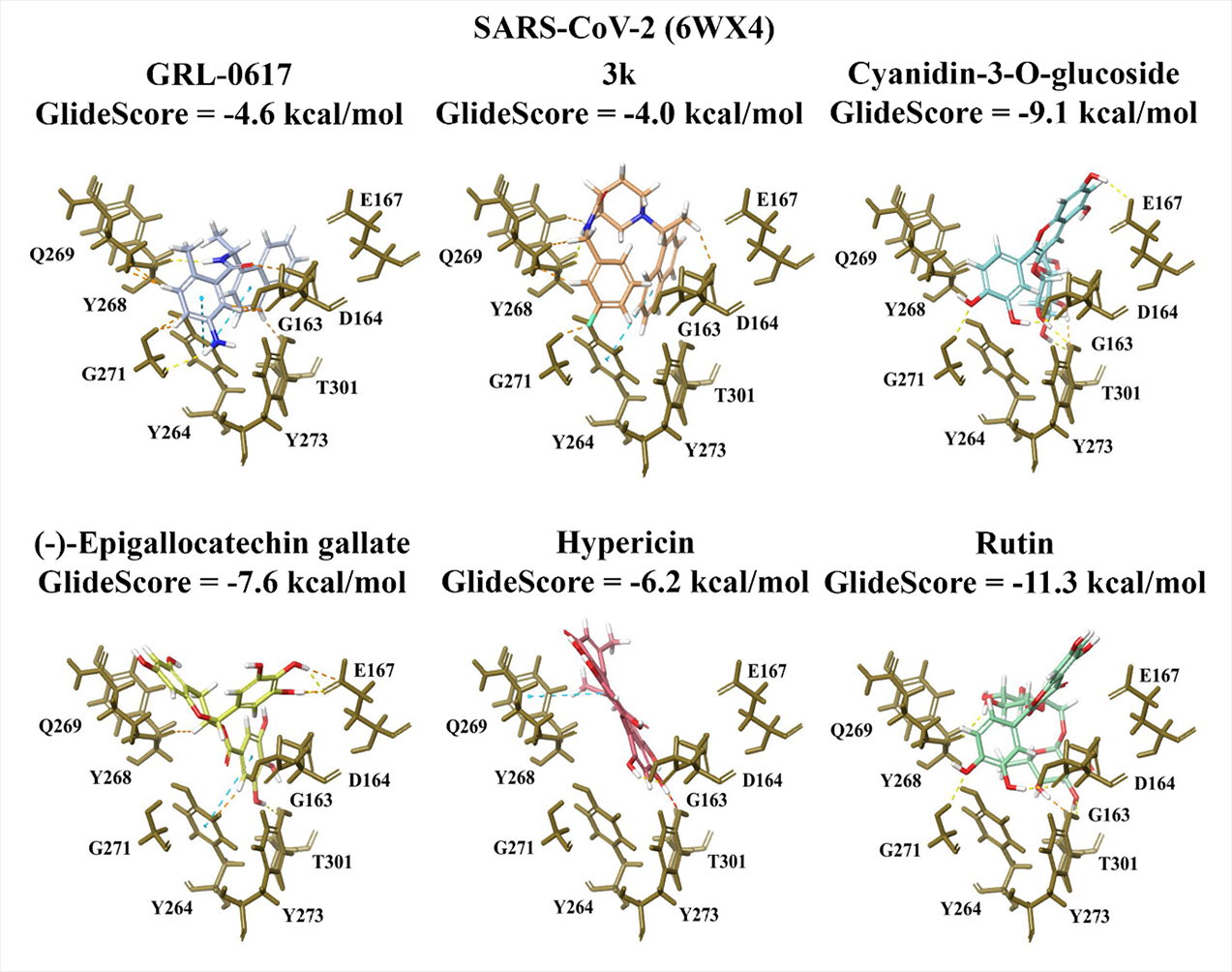

Supplement: Supplementary Figure 5 — Molecular docking results of the naphthalene-based inhibitors and dietary compounds for the SARS-CoV-2 PLpro structure 6wx4. [file Image_5.TIF]

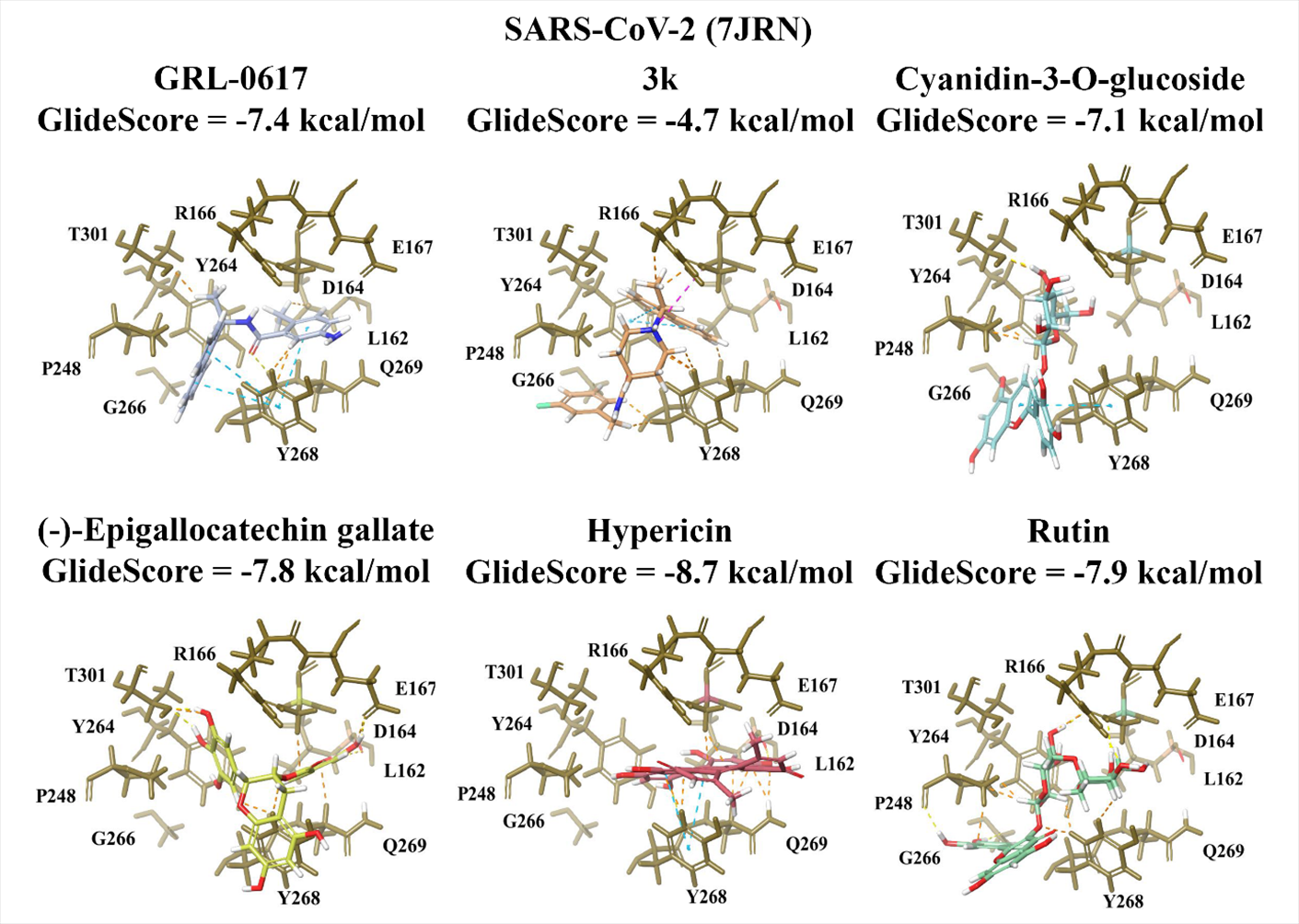

Supplement: Supplementary Figure 6 — Molecular docking results of the naphthalene-based inhibitors and dietary compounds for the SARS-CoV-2 PLpro structure 7jrn. [file Image_6.TIF]
