# Supplementary material for: Identification of Small Molecule Inhibitors of the Deubiquitinating Activity of the SARS-CoV-2 Papain-Like Protease: in silico Molecular Docking Studies and in vitro Enzymatic Activity Assay
Source: Front Chem. 2020 Dec 8;8:623971. doi: 10.3389/fchem.2020.623971 (PMC7753156; doi:10.3389/fchem.2020.623971)
Supplement: Supplementary Table 1 — PrankWeb conservation analysis results for the SARS-CoV-2 (6xaa), SARS-CoV (4mm3), and MERS-CoV PLpro (4rf0). [file Table_1.DOCX]

**Table S1.** PrankWeb conservation analysis results for the SARS-CoV-2 (6xaa), SARS-CoV (4mm3) and MERS-CoV PL^pro^ (4rf0).

| **Pocket** | **Ligandability Score** | **Residues** |
| --- | --- | --- |
| SARS-CoV-2 (Pocket 2) | 3.2 | 163, 164, 166, 208, 246, 248, 264, 268, 273, 301, 302 |
| SARS-CoV  (Pocket 3) | 3.3 | 164, 165, 167, 209, 249, 265, 274, 302, 303 |
| MERS-CoV  (Pocket 2) | 2.1 | 1645, 1646, 1648, 1729, 1731, 1750, 1754, 1756, 1758, 1760, 1789 |
