# Supplementary material for: Identification of Small Molecule Inhibitors of the Deubiquitinating Activity of the SARS-CoV-2 Papain-Like Protease: in silico Molecular Docking Studies and in vitro Enzymatic Activity Assay
Source: Front Chem. 2020 Dec 8;8:623971. doi: 10.3389/fchem.2020.623971 (PMC7753156; doi:10.3389/fchem.2020.623971)
Supplement: Supplementary Table 2 — Blind docking of the naphthalene-based inhibitors and dietary compounds to the SARS-CoV-2 (6xaa), SARS-CoV (4mm3), and MERS-CoV (4rf0) PLpro structures. [file Table_2.DOCX]

**Table S2.** Blind docking of the naphthalene-based inhibitors and dietary compounds to the SARS-CoV-2 (6xaa), SARS-CoV (4mm3) and MERS-CoV (4rf0) PL^pro^ structures.

| **Structure** | **Ligand** | **Pose** | **Binding Affinity (kcal/mol)** |
| --- | --- | --- | --- |
| 6xaa | GRL-0617 | 1 | -8.0 |
|  |  | 2 | -7.9 |
|  |  | 4 | -7.6 |
|  |  | 5 | -7.6 |
|  |  | 6 | -7.6 |
|  |  | 10 | -7.5 |
|  |  | 11 | -7.5 |
|  |  | 12 | -7.5 |
|  |  | 16 | -7.3 |
|  |  | 17 | -7.3 |
|  |  | 18 | -7.3 |
|  |  | 19 | -7.1 |
|  | 3k | 4 | -7.5 |
|  |  | 11 | -7.3 |
|  |  | 15 | -7.2 |
|  |  | 16 | -7.2 |
|  |  | 20 | -7.0 |
|  | Cyanidin-3-O-glucoside | 1 | -7.4 |
|  |  | 2 | -7.3 |
|  |  | 3 | -7.1 |
|  |  | 4 | -6.9 |
|  |  | 5 | -6.9 |
|  |  | 7 | -6.8 |
|  |  | 8 | -6.7 |
|  |  | 12 | -6.6 |
|  |  | 14 | -6.6 |
|  |  | 16 | -6.5 |
|  |  | 17 | -6.5 |
|  |  | 18 | -6.5 |
|  | (-)-Epigallocatechin gallate | 1 | -8.1 |
|  |  | 2 | -8.1 |
|  |  | 3 | -8.1 |
|  |  | 4 | -8.1 |
|  |  | 5 | -8.0 |
|  |  | 6 | -8.0 |
|  |  | 7 | -7.9 |
|  |  | 8 | -7.9 |
|  |  | 9 | -7.9 |
|  |  | 10 | -7.6 |
|  |  | 11 | -7.5 |
|  |  | 12 | -7.5 |
|  |  | 13 | -7.5 |
|  |  | 14 | -7.5 |
|  |  | 15 | -7.5 |
|  |  | 16 | -7.5 |
|  |  | 17 | -7.5 |
|  |  | 18 | -7.4 |
|  |  | 19 | -7.4 |
|  | Hypericin | 1 | -8.5 |
|  |  | 2 | -8.5 |
|  |  | 7 | -8.2 |
|  |  | 8 | -8.2 |
|  |  | 9 | -8.2 |
|  |  | 10 | -8.2 |
|  |  | 13 | -8.1 |
|  |  | 14 | -8.1 |
|  |  | 15 | -8.1 |
|  |  | 16 | -8.1 |
|  |  | 17 | -8.0 |
|  |  | 18 | -7.9 |
|  |  | 19 | -7.9 |
|  |  | 20 | -7.9 |
|  | Rutin | 1 | -7.6 |
|  |  | 2 | -7.5 |
|  |  | 4 | -7.3 |
|  |  | 5 | -7.2 |
|  |  | 6 | -7.2 |
|  |  | 7 | -7.1 |
|  |  | 8 | -7.1 |
|  |  | 9 | -7.1 |
|  |  | 10 | -7.0 |
|  |  | 11 | -7.0 |
|  |  | 12 | -7.0 |
|  |  | 13 | -7.0 |
|  |  | 15 | -7.0 |
|  |  | 16 | -7.0 |
|  |  | 18 | -6.9 |
|  |  | 19 | -6.8 |
| 4mm3 | GRL-0617 | 1 | -8.3 |
|  |  | 2 | -7.5 |
|  |  | 3 | -7.4 |
|  |  | 4 | -7.3 |
|  |  | 7 | -7.1 |
|  |  | 9 | -7.0 |
|  |  | 10 | -7.0 |
|  |  | 11 | -7.0 |
|  |  | 14 | -6.9 |
|  |  | 15 | -6.9 |
|  |  | 18 | -6.8 |
|  | 3k | 11 | -8.2 |
|  |  | 15 | -7.8 |
|  | Cyanidin-3-O-glucoside | 1 | -7.5 |
|  |  | 2 | -7.2 |
|  |  | 3 | -7.0 |
|  |  | 4 | -6.9 |
|  |  | 6 | -6.6 |
|  |  | 7 | -6.5 |
|  |  | 10 | -6.4 |
|  |  | 11 | -6.4 |
|  |  | 12 | -6.4 |
|  |  | 19 | -6.2 |
|  | (-)-Epigallocatechin gallate | 1 | -7.6 |
|  |  | 2 | -7.6 |
|  |  | 7 | -7.5 |
|  |  | 8 | -7.5 |
|  |  | 15 | -7.1 |
|  |  | 19 | -6.9 |
|  | Rutin | 1 | -7.4 |
|  |  | 3 | -7 |
|  |  | 4 | -7 |
|  |  | 6 | -6.9 |
|  |  | 7 | -6.9 |
|  |  | 10 | -6.8 |
|  |  | 11 | -6.8 |
|  |  | 12 | -6.8 |
|  |  | 16 | -6.7 |
|  |  | 18 | -6.6 |
|  |  | 19 | -6.6 |
|  |  | 20 | -6.6 |
|  | Hypericin | 5 | -8.0 |
|  |  | 6 | -8.0 |
|  |  | 16 | -7.6 |
|  |  | 17 | -7.6 |
| 4rf0 | GRL-0617 | 2 | -7.5 |
|  |  | 4 | -7.4 |
|  |  | 5 | -7.4 |
|  |  | 7 | -7.3 |
|  |  | 8 | -3.5 |
|  |  | 10 | -7.1 |
|  |  | 12 | -7.0 |
|  |  | 14 | -7.0 |
|  |  | 16 | -7.0 |
|  |  | 17 | -7.0 |
|  |  | 20 | -6.9 |
|  | 3k | 1 | -8.5 |
|  |  | 2 | -8.5 |
|  |  | 4 | -8.3 |
|  |  | 6 | -8.3 |
|  |  | 7 | -8.3 |
|  |  | 9 | -8.1 |
|  |  | 10 | -8.1 |
|  |  | 11 | -8.0 |
|  |  | 13 | -8.0 |
|  |  | 16 | -7.8 |
|  |  | 19 | -7.7 |
|  | Cyanidin-3-O-glucoside | 1 | -7.4 |
|  |  | 2 | -7.3 |
|  |  | 3 | -7.2 |
|  |  | 4 | -7.1 |
|  |  | 5 | -7.0 |
|  |  | 7 | -6.8 |
|  |  | 9 | -6.7 |
|  |  | 10 | -6.7 |
|  |  | 11 | -6.7 |
|  |  | 12 | -6.7 |
|  | (-)-Epigallocatechin gallate | 1 | -6.7 |
|  |  | 2 | -6.7 |
|  |  | 3 | -6.6 |
|  |  | 4 | -6.6 |
|  |  | 5 | -6.6 |
|  |  | 6 | -6.6 |
|  |  | 7 | -6.6 |
|  |  | 8 | -6.6 |
|  |  | 9 | -6.6 |
|  |  | 10 | -6.6 |
|  |  | 11 | -6.5 |
|  |  | 12 | -6.5 |
|  |  | 13 | -6.3 |
|  |  | 14 | -6.3 |
|  |  | 15 | -6.3 |
|  |  | 16 | -6.3 |
|  |  | 17 | -6.3 |
|  |  | 18 | -6.2 |
|  |  | 19 | -6.1 |
|  | Hypericin | 5 | -8.4 |
|  |  | 6 | -8.4 |
|  |  | 11 | -7.8 |
|  |  | 14 | -7.7 |
|  |  | 17 | -7.6 |
|  |  | 18 | -7.6 |
|  | Rutin | 1 | -7.3 |
|  |  | 3 | -7.2 |
|  |  | 4 | -7.1 |
|  |  | 5 | -6.9 |
|  |  | 6 | -6.9 |
|  |  | 8 | -6.7 |
|  |  | 9 | -6.7 |
|  |  | 11 | -6.6 |
|  |  | 15 | -6.5 |
|  |  | 16 | -6.5 |
|  |  | 17 | -6.5 |
|  |  | 19 | -6.4 |
