# Supplementary material for: Identification of Small Molecule Inhibitors of the Deubiquitinating Activity of the SARS-CoV-2 Papain-Like Protease: in silico Molecular Docking Studies and in vitro Enzymatic Activity Assay
Source: Front Chem. 2020 Dec 8;8:623971. doi: 10.3389/fchem.2020.623971 (PMC7753156; doi:10.3389/fchem.2020.623971)
Supplement: Supplementary Table 4 — The GlideScores (kcal/mol) of the naphthalene-based inhibitors and dietary compounds for the SARS-CoV-2, SARS-CoV, and MERS-CoV crystal structures with ubiquitin are listed. [file Table_4.DOCX]

**Table S4.** The GlideScores (kcal/mol) of the naphthalene-based inhibitors and dietary compounds for the SARS-CoV-2, SARS-CoV and MERS-CoV crystal structures with ubiquitin are listed.

| **Compound** | **SARS-CoV-2: GlideScore (kcal/mol)** | **SARS-CoV: GlideScore (kcal/mol)** | **MERS-CoV: GlideScore (kcal/mol)** |
| --- | --- | --- | --- |
| GRL-0617 | -3.5 | -3.7 | -2.2 |
| 3k | -2.8 | -3.4 | -1.9 |
| (-)-Epigallocatechin gallate | -4.7 | -5.8 | -7.4 |
| Rutin | -6.5 | -8.1 | -7.0 |
| Hypericin | - | - | - |
| Cyanidin-3-O-glucoside | -6.6 | -7.2 | -7.8 |
