# Supplementary material for: Identification of Small Molecule Inhibitors of the Deubiquitinating Activity of the SARS-CoV-2 Papain-Like Protease: in silico Molecular Docking Studies and in vitro Enzymatic Activity Assay
Source: Front Chem. 2020 Dec 8;8:623971. doi: 10.3389/fchem.2020.623971 (PMC7753156; doi:10.3389/fchem.2020.623971)
Supplement: Supplementary Table 5 — The docking scores and RMSD values for the top-ranked PLpro-ubiquitin complexes from the HDOCK server are provided. [file Table_5.DOCX]

**Table S5.** The docking scores and RMSD values for the PL^pro^-ubiquitin complexes from the HDOCK server are provided.

| **Structure** | **Compound** | **Rank** | **Docking Score** | **Ligand RMSD (**Å) |
| --- | --- | --- | --- | --- |
| SARS-CoV-2 (6XAA) | No ligand | 1 | -330.3 | 0.4 |
|  | 3k | 1 | -215.8 | 21.0 |
|  | (-)-Epigallocatechin gallate | 1 | -205.6 | 21.2 |
|  | GRL-0617 | 1 | -203.2 | 38.8 |
|  | Hypericin | 1 | -209.5 | 23.5 |
|  | Rutin | 1 | -239.4 | 12.9 |
|  | Cyanidin-3-O-glucoside | 1 | -208.1 | 21.0 |
| SARS-CoV (4MM3) | No ligand | 1 | -295.7 | 0.6 |
|  | 3k | 1 | -205.6 | 15.7 |
|  | (-)-Epigallocatechin gallate | 1 | -203.5 | 23.1 |
|  | GRL-0617 | 1 | -203.2 | 23.2 |
|  | Hypericin | 1 | -204.0 | 23.03 |
|  | Rutin | 1 | -223.0 | 7.80 |
|  | Cyanidin-3-O-glucoside | 1 | -207.6 | 7.63 |
| MERS-CoV (4RF0) | No ligand | 1 | -389.3 | 0.5 |
|  | 3k | 1 | -244.9 | 23.2 |
|  | (-)-Epigallocatechin gallate | 1 | -247.1 | 23.1 |
|  | GRL-0617 | 1 | -253.1 | 23.9 |
|  | Hypericin | 1 | -234.6 | 21.9 |
|  | Rutin | 1 | -246.3 | 23.8 |
|  | Cyanidin-3-O-glucoside | 1 | -242.6 | 23.8 |
